# Increased Susceptibility to *Salmonella* Infection in Systemic Lupus Erythematosus Compared with Other Systemic Autoimmune Diseases: Insights from a Retrospective Cohort Study from the Largest Health Care System in Taiwan

**DOI:** 10.3390/life15101522

**Published:** 2025-09-26

**Authors:** Chen-Ying Wei, Han-Hua Yu, Pei-Yi Cheng, Yen-Fu Chen

**Affiliations:** 1 Division of Chinese Internal Medicine, Center for Traditional Chinese Medicine, Chang Gung Memorial Hospital, Taoyuan 333, Taiwan; hedywei@hotmail.com; 2 Division of Rheumatology, Allergy and Immunology, Linkou Chang Gung Memorial Hospital, Taoyuan 333, Taiwan; hanhua.yu.9@gmail.com; 3 College of Medicine, Chang Gung University, Taoyuan 333, Taiwan; 4 Center for Big Data Analytics and Statistics, Linkou Chang Gung Memorial Hospital, Taoyuan 333, Taiwan; peiyizheng47@gmail.com

**Keywords:** systemic lupus erythematosus, systemic autoimmune disease, *Salmonella*

## Abstract

Systemic lupus erythematosus (SLE) and other systemic autoimmune rheumatic diseases (SARDs) require long-term immunosuppressive therapy, placing patients at increased risk of infection. *Salmonella* species are particularly concerning due to their invasiveness and potential link to autoimmune activation, notably in SLE. This study aimed to compare the risk of culture-confirmed *Salmonella* infection between SLE and other SARDs, based on data from the Chang Gung Research Database between 2005 and 2020. After propensity score matching, 3537 patients per group were analyzed. Patients with SLE had a higher incidence of *Salmonella* infection compared with those with other SARDs (0.54 vs. 0.17 per 1000 person-years), with a significantly greater cumulative incidence (log-rank *p* < 0.01). The adjusted hazard ratio (HR) for *Salmonella* infection in SLE was 2.47 (95% confidence interval (CI): 0.95–6.38), and the competing risk model confirmed a significant association (sub-distribution HR 2.58, 95% CI: 1.06–6.29, *p* = 0.04). Among SLE patients, lymphopenia was the only independent predictor of *Salmonella* infection (adjusted HR 3.98, 95% CI: 1.83–8.68, *p* < 0.001). Bloodstream infections were most common (70%), and serogroup D was the predominant strain (80%). These results suggest patients with SLE face higher *Salmonella* risk than other SARDs, especially those with lymphopenia, underscoring the need for targeted surveillance and preventive strategies.

## 1. Introduction

Systemic lupus erythematosus (SLE) and other systemic autoimmune rheumatic diseases (SARDs)—including Sjögren’s syndrome, rheumatoid arthritis, systemic sclerosis, dermatomyositis, and polymyositis—are chronic, immune dysregulation disorders with multi-organ involvements. These conditions often necessitate long-term immunosuppressive or immunomodulatory therapy to control disease activity and prevent irreversible damages [1,2].

While advances in immunosuppressive treatment have improved disease control and survival, infection remain a leading cause of morbidity and mortality in these populations [3,4]. For example, among various immunosuppressive strategies, tumor necrosis factor-alpha inhibitors have been identified as important risk factors for opportunistic infections caused by intracellular pathogens such as *Salmonella* and *Mycobacteria*, as well as certain fungi [5].

Among these opportunistic infections, *Salmonella* species are of particular concern in immunocompromised adults due to their heightened risk of invasive disease manifestations such as primary bacteremia and recurrent infections, often associated with increased mortality [6]. Notably, emerging evidence also suggests a possible bidirectional relationship between *Salmonella* infection and autoimmunity. A nationwide, population-based case–control study in Taiwan reported that individuals with a history of nontyphoidal *Salmonella* infection had a significantly higher risk of developing SLE, with an adjusted odds ratio exceeding 7 in both conventional and propensity score-matched analyses [7]. This suggests that, in addition to being a common opportunistic pathogen in patients with established SLE, nontyphoidal *Salmonella* infection may also act as a potential environmental trigger for autoimmune activation through dysregulated inflammatory pathways.

Although *Salmonella* infection has long been recognized as a clinically important opportunistic infection in patients with SLE [8,9,10]. A single-center study by Abramson et al. identified SLE as the most common underlying condition among patients with *Salmonella* bacteremia, accounting for 20% of cases, a striking overrepresentation compared to non-*Salmonella* bacteremia [11]. These observations suggest that SLE patients may be particularly susceptible due to both disease-related immune dysregulation and immunosuppressive therapy. However, it remains unclear whether this heightened vulnerability is unique to SLE or reflective of a broader risk pattern across other SARDs, many of which similarly require prolonged immunomodulatory treatment.

Importantly, a large epidemiological analysis from the Chinese Rheumatism Data Center revealed that SLE is associated with a standardized mortality ratio (SMR) of 3.5—higher than rheumatoid arthritis (SMR 0.96) and Sjogren’s syndrome (SMR 1.53) [12]. Adding to the complexity, nationwide studies in Taiwan have demonstrated that SLE not only imposes a substantial clinical burden, with highest mortality concentrated in newly diagnosed young patients [13], but also represents a significant economic burden, with per-patient societal costs reaching over 33,000 USD annually and infections constituting a major driver of health care utilization [14].

To date, no large-scale cohort study has compared the risk of *Salmonella* infection across SLE and other SARDs. Therefore, the present study aimed to fill this knowledge gap by evaluating the hazard ratio of *Salmonella* infection in SLE versus other SARDs utilizing the Chang Gung Research Database (CGRD)—a database from the largest health care system in Taiwan—and identify key clinical predictors, including immune parameters and medication exposures.

## 2. Materials and Methods

### 2.1. Data Source

We utilized the CGRD in Taiwan to compare the risk of *Salmonella* infection between the SLE patients and other SARDs patients. The definition of SLE was determined according to catastrophic illness record with code 710.0 of the International Classification of Disease, Ninth Revision, Clinical Modification (ICD-9-CM) or code M32 of the International Classification of Disease, Tenth Revision, Clinical Modification (ICD-10-CM). Other SARDs included Sjogren’s syndrome, rheumatoid arthritis, dermatomyositis/polymyositis, and systemic sclerosis, which were also defined according to catastrophic illness record. The ICD-9-CM and ICD-10-CM codes for Sjogren’s syndrome are 710.2 and M35.0, respectively. The ICD-9-CM codes for rheumatoid arthritis are 714.0 and 714.30–714.33, and the ICD-10-CM codes are M05.70–M06.09, M06.20–M06.39, M06.8, M06.9, and M08. The ICD-9-CM codes for dermatomyositis/polymyositis are 710.3 and 710.4, and the ICD-10-CM codes are M33.2, M33.00-M33.19, M33.9, and M36.0. The ICD-9-CM and ICD-10-CM codes for systemic sclerosis are 710.1 and M34, respectively.

### 2.2. Ethical Statement

This study was approved by the Institutional Review Board of Chang Gung Medical Foundation (IRB No. 202202074B0C6004). The IRB approved the waiver of informed consent since de-identified data was used.

### 2.3. Study Design

**Figure 1:**
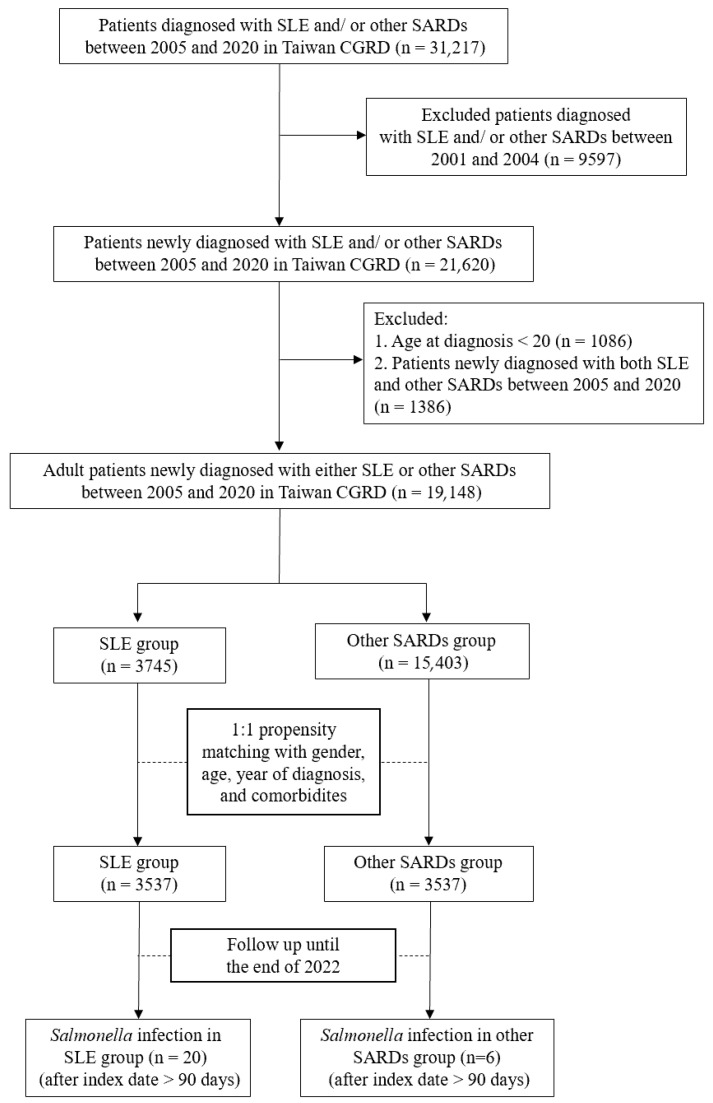
Flow chart of the study. Abbreviations: SLE, systemic lupus erythematosus; SARDs, systemic autoimmune rheumatic diseases.

We initially included 31,217 patients diagnosed with SLE or other SARDs between 2005 and 2020. After excluding patients diagnosed between 2001 and 2004, those under 20 years of age at diagnosis, and those diagnosed with both SLE and other SARDs during the study period, a total of 19,148 adult patients remained. These were categorized into the newly diagnosed SLE group (*n* = 3745) and the newly diagnosed other SARDs group (*n* = 15,403). The index date for each patient was defined as the date of their initial diagnosis of SLE or other SARDs. To ensure comparability between groups, we conducted a 1:1 propensity score matching based on sex, age, year of diagnosis, and comorbidities. By leaving only patients new diagnosed between 2005 and 2020 and matching with the year of first diagnosis, the effect of patients with different lengths of the course of SLE or other SARDs might be mitigated. This yielded a final matched cohort of 7074 patients (3537 in each group), who were followed up until the end of 2022. Figure 1 presents the study flowchart.

### 2.4. Study Variables

Our main outcome was a *Salmonella* infection event during the 18 years follow-up period, which was defined as a bacterial culture result with *Salmonella* in the blood, urine, sputum, ascites, or stool. Comorbidities included chronic diseases with a potential effect on our outcome, such as diabetes mellitus, chronic kidney disease, liver cirrhosis, and lymphoproliferative disorder/cancer. One inpatient hospitalization or two outpatient visits due to any of the chronic diseases mentioned above within 1 year before the index date was considered comorbidity. The criterion for the use of corticosteroids, conventional synthesized disease-modifying antirheumatic drugs (DMARDs), and JAK inhibitors was a medication prescription period of no less than 90 days in the year preceding the index date. The criterion for the use of biological DMARDs was used at least once in the year preceding the index date.

### 2.5. Statistical Analysis

Age was presented as mean and standard deviation, and Student’s t-test was used for group comparisons. Categorical data were presented as numbers and percentages, and the chi-squared test was used for group comparisons. A standardized difference was also used for group comparisons to demonstrate the effect size. To compare the risk of *Salmonella* infection between the SLE and other SARDs groups, we executed the Cox proportional hazard regression model adjusted for factors including age, sex, comorbidities, and medications. In addition, the Cox proportional hazard regression model was also used to further analyze the risk factors of *Salmonella* infection among the SLE patients. We included risk factors with *p*-value < 0.2 in the stepwise selection into the multivariate analysis.

All statistical analyses were utilized by SAS 9.4 (SAS Institute, Cary, NC, USA). All statistical tests were 2-sided, and a *p*-value < 0.05 being considered statistically significant.

## 3. Results

### 3.1. Baseline Demographic Characteristics

Between 1 January 2005, and 31 December 2020, 19,148 patients with either SLE or other SARDs were included. After matching by propensity score, this cohort comprised 3537 SLE patients and an equal number of patients with other SARDs, ensuring a balanced comparison between the two groups. We analyzed baseline demographic and clinical characteristics between the SLE and other SARDs groups using standardized differences, as shown in Table 1. The effect sizes of age, sex and comorbidities for all characteristics were below 0.2. According to prior studies on standardized difference, the differences in these baseline characteristics between the two groups in this study were negligible [15,16]. However, in terms of drug use, several DMARDs showed notable imbalances in usage between groups: azathioprine (6.73% in SLE vs. 2.21%, effect size = 0.22), methotrexate (0.48% vs. 16.03%, effect size = 0.59), and sulfasalazine (0.40% vs. 11.17%, effect size = 0.50).

### 3.2. Incidence and Risk Comparison of Salmonella Infection in SLE and Other SARDs

**Figure 2:**
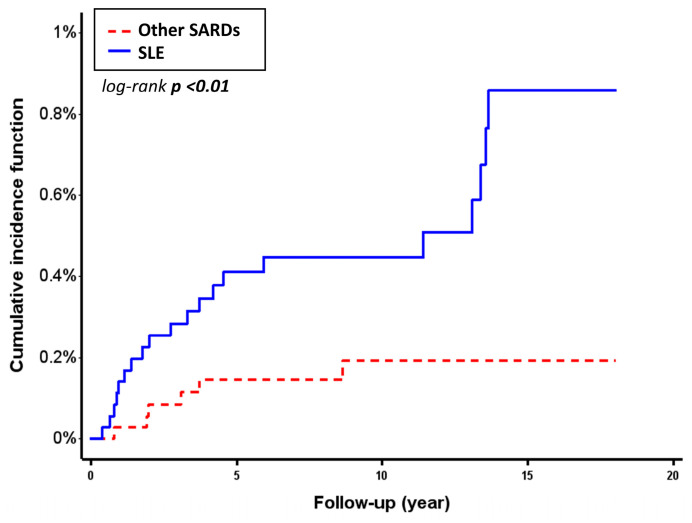
Cumulative incidence of *Salmonella* infection over 18 years in SLE and other SARDs. Abbreviations: SARDs, systemic autoimmune rheumatic diseases; SLE, systemic lupus erythematosus.

Patients with SLE had a higher incidence of *Salmonella* infection compared to those with other SARDs. The incidence rate of *Salmonella* infection in the SLE group was 0.54 per 1000 person-years (95% confidence interval (CI): 0.31–0.78), whereas it was 0.17 per 1000 person-years (95% CI: 0.03–0.30) in the comparison group. Kaplan–Meier analysis showed a significantly higher cumulative incidence of *Salmonella* infection in the SLE group (log-rank *p* < 0.01) (Figure 2.)

At the end of the 18-year-follow-up, the Cox proportional hazards model showed a crude hazard ratio (HR) of 3.28 (95% CI: 1.32–8.18) and an adjusted HR 2.47 (95% CI: 0.95–6.38, *p* = 0.06) for *Salmonella* infection in comparison of the SLE group with the other SARDs group. Furthermore, a competing risk analysis was performed with death as a competing event, yielding a sub-distribution hazard ratio of 2.58 (95% CI: 1.06–6.29, *p* = 0.04), confirming an independently increased risk of *Salmonella* infection in patients with SLE. These findings are summarized in Table 2.

### 3.3. Risk Factors for Salmonella Infection in the Patients with SLE

In the univariate Cox proportional hazards analysis, several factors were significantly associated with an increased risk of *Salmonella* infection among patients with SLE, including male sex (HR 2.55, 95% CI: 1.07–6.07, *p* = 0.03), lymphoproliferative disorder or cancer (HR 3.08, 95% CI: 1.06–8.94, *p* = 0.04), leukopenia (HR 2.67, 95% CI: 1.19–6.02, *p* = 0.02), lymphopenia (HR 3.98, 95% CI: 1.83–8.68, *p* < 0.001), decreased C3 (HR 2.48, 95% CI: 1.13–5.47, *p* = 0.02), proteinuria (HR 4.06, 95% CI: 1.39–11.92, *p* = 0.01), and hepatitis (HR 3.17, 95% CI: 1.20–8.41, *p* = 0.02) (Table 3).

Among DMARDs, azathioprine (HR 3.03, 95% CI: 0.91–10.12, *p* = 0.07), mycophenolic acid (HR 3.91, 95% CI: 0.53–28.99, *p* = 0.19), and cyclosporin (HR 4.22, 95% CI: 0.57–31.15, *p* = 0.16) demonstrated non-significant but suggestive trends toward increased risk.

Variables with a *p*-value < 0.2 in the univariate analysis were subsequently included in a stepwise multivariate Cox proportional hazards regression model. In the final model, only lymphopenia remained independently associated with a significantly higher risk of *Salmonella* infection (HR 3.98, 95% CI: 1.83–8.68, *p* < 0.001).

### 3.4. Serogroup and Sample Source of Salmonella in SLE

**Figure 3:**
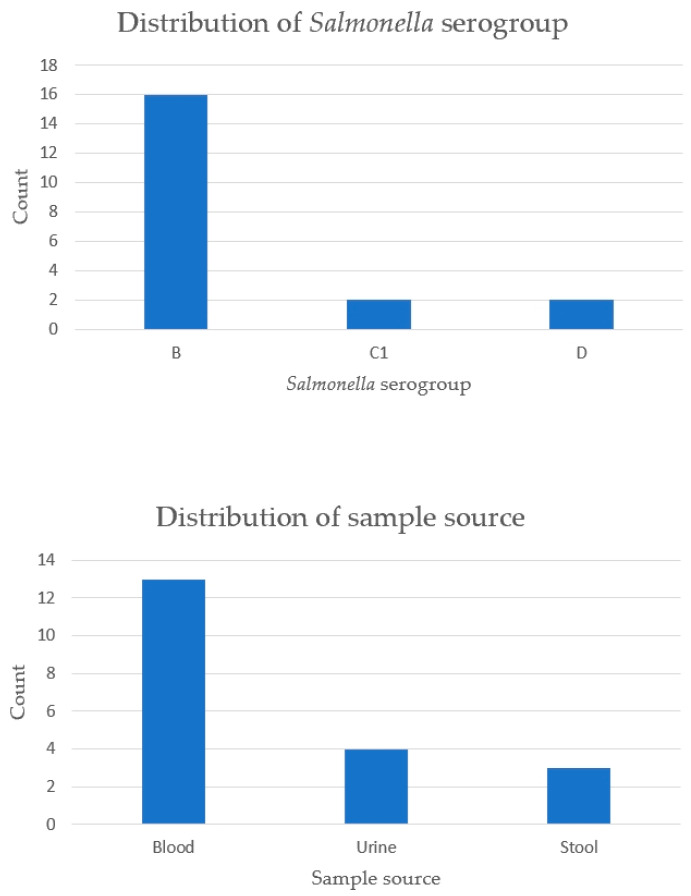
Serogroup and sample source of *Salmonella* infection among SLE patients. Abbreviation: SLE, systemic lupus erythematosus.

Among the 20 SLE patients with *Salmonella* infection, serogroup D was the most frequently identified strain (80%), followed by B (15%) and C1 (10%). The most common specimen source was blood (70%), followed by urine (20%) and stool (15%). This distribution suggested that bloodstream infections with serogroup D are the predominant clinical presentation in this population (Figure 3.)

## 4. Discussion

This large-scale retrospective cohort study demonstrated a significant higher risk of *Salmonella* infection in SLE patients compared to other SARDs. While several clinical factors were associated with increased risk among the SLE patients in the univariate analysis, lymphopenia emerged as the most robust independent predictor after adjusting for other covariates.

Infection is a major contributor to both morbidity and mortality in patients with SLE, accounting for an estimated 30–50% of overall disease-related complications worldwide [17,18]. An analysis from the United Kingdom revealed a striking rise in hospitalizations for severe infections among SLE patients, with the rate surging more than 12 times between 1996 and 2001 [3].

Among the causative pathogens of infection, *Salmonella* infection has long been recognized as a clinically important opportunistic infection in patients with SLE [9,11]. Previous studies have reported that patients with SLE are disproportionately vulnerable to *Salmonella* infection compared to general population. In a retrospective study of adult patients at Bellevue Hospital from 1975 to 1982, Abramson et al. found that SLE was the most frequent underlying disease among those with *Salmonella* bacteremia, accounting for 20% of cases, compared to only 0.54% in non-*Salmonella* Gram-negative bacteremia [11]. The disproportionately high rate of Salmonella bacteremia among SLE patients observed in this study may be attributed to their widespread use of immunosuppressive agents and underlying humoral immune defects, such as hypocomplementemia, both of which impair host defense mechanisms against invasive pathogens [11].

Exposure to *Salmonella* is associated with the development of SLE, which is possibly caused by releasing bacterial amyloid curli/DNA complexes [7,19,20]. Previous study also indicated that nontyphoidal *Salmonella enterica* infections, particularly those caused by *S. typhimurium* and *S. enteritidis*, may contribute to elevated autoantibody levels. Patients demonstrated significantly increased titers of autoantibodies during both the acute and convalescent phases of infection [21]. On the other hand, there is a high association between non-typhoidal salmonellosis and SLE, especially among patients with active disease [22]. As a result, development of the autoantibodies due to exposure to *Salmonella* among SLE patients could possibly increase disease activity and then result in severe *Salmonella* infection. As one of the opportunistic infection pathogens, *Salmonella* in SLE patients might progress to clinically significant infection by the vicious cycle of this bidirectional relationship.

While previous studies have primarily focused on the heightened risk of *Salmonella* infection in SLE patients compared to the general population, less is known about how this risk compares to that in patients with other SARDs, who also commonly receive immunosuppressive treatment. By directly comparing SLE with other SARDs, our study helps clarify whether this vulnerability is disease-specific or merely reflective of generalized immune dysfunction across autoimmune diseases.

To further characterize disease-specific immune vulnerability, we investigated potential predictors of *Salmonella* infection among SLE patients. In the univariate Cox proportional hazards analysis, male sex was significantly associated with an increased risk of *Salmonella* infection. Compared with female SLE patients, male SLE patients have higher disease activity and more severe organ damage and may require more aggressive treatment [23,24], which might explain the greater susceptible to infection. In addition, previous studies showed hematologic involvement was associated with serious infections in SLE patients and antimalarial use was protective [25,26]. Although our study did not reveal significant effects in hydroxychloroquine use, lymphopenia stood out as the only independent risk factor in our multivariate analysis—a finding that aligns with prior observations.

In a retrospective study conducted by Tsao et al. involving 31 hospitalized SLE patients with *Salmonella* infection, lymphopenia was observed in 74.2% of cases, suggesting a high prevalence of T-cell immune dysfunction in this population [27]. Further supporting the predictive role of lymphopenia, a retrospective case–control study by Merayo-Chalico et al. investigated 167 SLE patients and found that lymphopenia was independently associated with a significantly higher risk of severe infections, with an odds ratio of 5.2 (95% CI: 2.39–11.3), even after adjusting for other variables such as disease activity and immunosuppressive therapy [28].

Lymphopenia is a frequent hematologic abnormality in SLE, affecting T lymphocytes—particularly CD4^+^ cells—more profoundly than B cells. Its mechanisms are multifactorial, involving increased apoptosis, complement-mediated cytolysis, and impaired lymphopoiesis [29]. Clinically, lymphopenia has been associated with disease activity, renal involvement, and immunosuppressive therapy. In a cross-sectional study of 124 SLE patients, lymphopenia was significantly associated with lupus nephritis, complement consumption, higher steroid dose, and cyclophosphamide use [30]. These findings suggest that lymphopenia may serve as a potential clinical marker of immunologic severity and renal involvement in patients with SLE.

Given that therapeutic regimens may influence infection susceptibility, we first compared immunosuppressive drug use between groups. Standardized difference analysis revealed notable imbalances in the use of DMARDs—particularly methotrexate, sulfasalazine, and azathioprine—between the SLE and other SARDs groups. These discrepancies likely reflect divergent therapeutic strategies tailored to the underlying disease pathology, disease activity, or organ involvement, and may in turn contribute to differential infection risk profiles across groups. To account for these potential confounding effects, we incorporated medication use, including specific DMARDs and corticosteroids, into our multivariate Cox proportional hazards model. This adjustment allowed us to isolate the association between disease groups (SLE vs. other SARDs) and *Salmonella* infection risk, independent of variations in immunosuppressive treatment regimens.

Beyond assessing overall infection risk, we further analyzed the distribution of serogroups and specimen sources of *Salmonella* isolates to better understand the microbiological profiles and infection sites in SLE patients. Compared to real-world evidence, the previous study by Yu et al. consistently identified bacteremia as the predominant clinical manifestation of *Salmonella* infection in SLE patients, with blood cultures yielding the highest proportion of positive isolates [31]. However, the distribution of *Salmonella* serogroups differed between the two cohorts. In our analysis, serogroup D accounted for the majority of infections (80%), followed by groups B (15%) and C1 (10%). In contrast, Yu et al. reported serogroup B as the most frequently isolated strain (57.2%), with no mention of serogroup D [31]. This discrepancy may reflect temporal changes in circulating *Salmonella* strains, regional epidemiological differences, or distinct patient immunologic backgrounds and treatment exposures.

Our study offers several strengths that enhance the validity of its findings. First, previous studies had largely concentrated on case-based descriptions of *Salmonella* infections in SLE, while our study directly compared incidence and risk with other SARDs. Second, outcome analyses were performed using both Cox proportional hazards regression and competing risk models, which accounted for death as a competing event. This dual modeling strategy strengthened the reliability of our risk estimates, particularly in the presence of differential mortality risks. Third, we applied a stepwise variable selection approach in our multivariate modeling, allowing for dynamic inclusion of predictors based on statistical significance. This method helped address multicollinearity and interaction among variables—an important consideration in observational cohorts involving complex immune-mediated diseases and overlapping treatment exposures. Finally, a key strength of this study lies in the use of the CGRD, which provided access to detailed laboratory test results. This enabled us to incorporate objective immune and inflammatory biomarkers into the risk models, offering greater specificity than many administrative databases that lack laboratory data.

Several limitations should be considered in this study. First, medication exposure was assessed at baseline only, and potential time-varying effects throughout the follow-up period could not be fully captured. Second, despite multivariate adjustments, residual confounding may persist. Unmeasured behavioral or environmental factors—such as dietary habits, hygiene practices, and recent travel history—might have influenced individual infection risk but were not available in the database.

Building upon these strengths, and acknowledging the limitations of the study, our findings may carry important clinical implications. Clinicians may consider maintaining a heightened awareness of the potential for *Salmonella* infection in patients with SLE, particularly those with lymphopenia. Future studies could explore whether monitoring lymphocyte subsets might benefit high-risk subgroups. Moreover, prospective research incorporating dynamic immune profiling and microbiological data may provide further insights into host–pathogen interactions and help refine infection risk assessment in this population.

## Figures and Tables

**Table 1:** Baseline patient characteristics in the SLE and other SARDs groups.

	SLE (*n* = 3537)	Other SARDs (*n* = 3537)	*p*-Value ^a^	Standardized Difference ^b^
**Age**	42.93 ± 15.02	44.00 ± 14.31	<0.01	0.07
**Male**	445 (12.58)	445 (12.58)	1	0
**Comorbidity**				
Diabetes mellitus	166 (4.69)	216 (6.11)	0.01	0.06
Chronic kidney disease	522 (14.76)	444 (12.55)	0.01	0.06
Cirrhosis	64 (1.81)	63 (1.78)	0.93	<0.01
Lymphoproliferative disorder/cancer	187 (5.29)	219 (6.19)	0.10	0.04
**Drug**				
Steroids	1263 (35.71)	1047 (29.60)	<0.001	0.13
**DMARDS**				
Hydroxychloroquine	1040 (29.40)	1041 (29.43)	0.98	<0.001
Azathioprine	238 (6.73)	78 (2.21)	<0.001	0.22
Methotrexate	17 (0.48)	567 (16.03)	<0.001	0.59
Leflunomide	2 (0.06)	61 (1.72)	<0.001	0.18
Mycophenolic acid	78 (2.21)	12 (0.34)	<0.001	0.17
Cyclosporin	27 (0.76)	49 (1.39)	0.11	0.09
Cyclophosphamide	17 (0.48)	9 (0.25)	0.12	0.03
Sulfasalazine	14 (0.40)	395 (11.17)	<0.001	0.50
Tofacitinib	0 (0.00)	0 (0.00)	-	-
Baricitinib	0 (0.00)	0 (0.00)	-	-
Upadacitinib	0 (0.00)	0 (0.00)	-	-
Rituximab	7 (0.20)	8 (0.23)	0.80	0.01
Etanercept	1 (0.03)	13 (0.37)	<0.01	0.08
Adalimumab	0 (0.00)	7 (0.20)	0.02	0.06
Golimumab	0 (0.00)	2 (0.06)	0.50	0.03
Certolizumab	0 (0.00)	7 (0.20)	0.02	0.06
Infliximab	0 (0.00)	0 (0.00)	-	-
Tocilizumab	0 (0.00)	14 (0.40)	<0.001	0.09
Abatacept	0 (0.00)	3 (0.08)	0.25	0.04
Belimumab	0 (0.00)	0 (0.00)	-	-

^a^ Chi-squared test for categorical data and unpaired Student *t*-test for continuous data. ^b^ Calculation of effect size: mean for continuous data and percentage for categorical data. Abbreviations: DMARDs, disease-modifying antirheumatic drugs; SARDs, systemic autoimmune rheumatic diseases; SLE, systemic lupus erythematosus.

**Table 2:** Incidence and risk comparison of *Salmonella* infection in SLE and other SARDs.

	Number	Follow-Up (Years)	Person-Years of Follow-Up	Incidence Rate, Per 1000 Person-Years	Crude HR (95% CI)	Adjusted HR ^a^ (95% CI)	Competing Risk Regression Model ^b^ Adjusted HR ^a^ (95% CI)
SLE	20/3537	10.38 ± 4.75	36,703.71	0.54 (0.31–0.78)	3.28 (1.32–8.18)	2.47 (0.95–6.38)	2.58 (1.06–6.29)
Other SARDs	6/3537	10.19 ± 4.57	36,059.53	0.17 (0.03–0.30)	-	-	-
*p*-value	<0.01	-	-	-	0.01	0.06	0.04

^a^ Adjusted for age, sex, baseline comorbidities, and medications; ^b^ death as a competing event. Abbreviations: SARDs, systemic autoimmune rheumatic diseases; SLE, systemic lupus erythematosus; HR, hazard ratio; CI, confidence interval.

**Table 3:** Risk factors of *Salmonella* infection in patients with SLE.

**Variable**	**Univariate Analysis**		**Multivariate Analysis**	
	**HR (95% CI)**	***p*-Value**	**HR (95% CI)**	***p*-Value**
Age ≥ 50	0.86 (0.36–2.05)	0.74	-	
Male	2.55 (1.07–6.07)	0.03	-	
**Comorbidity**				
Diabetes mellitus	2.38 (0.72–7.94)	0.16	-	
Chronic kidney disease	1.08 (0.37–3.13)	0.89	-	
Cirrhosis	2.33 (0.32–17.16)	0.41	-	
Lymphoproliferative disorder/cancer	3.08 (1.06–8.94)	0.04	-	
**DMARDS**				
Hydroxychloroquine	1.30 (0.58–2.92)	0.53	-	
Azathioprine	3.03 (0.91–10.12)	0.07	-	
Mycophenolic acid	3.91 (0.53–28.90)	0.18	-	
Cyclosporin	4.22 (0.57–31.15)	0.16	-	
**Laboratory data**				
Leukopenia	2.67 (1.19–6.02)	0.02	-	
Lymphopenia	3.98 (1.83–8.68)	<0.001	3.98 (1.83–8.68)	<0.001
Decreased IgM	5.63 (0.76–41.56)	0.09	-	
Decreased C3	2.48 (1.13–5.47)	0.02	-	
Decreased C4	2.41 (1.02–5.74)	0.05	-	
Positivity of anti-dsDNA	1.02 (0.45–2.28)	0.97	-	
Increased ferritin	2.66 (0.63–11.28)	0.18	-	
Proteinuria	4.06 (1.39–11.92)	0.01	-	
Hepatitis	3.17 (1.20–8.41)	0.02	-	
Renal impairment	1.87 (0.71–4.96)	0.21	-	
Hypoalbuminemia	1.68 (0.68–4.19)	0.26	-	
Elevated ESR	0.37 (0.13–1.06)	0.06	-	

Abbreviations: SLE, systemic lupus erythematosus; IgM, Immunoglobulin M; C3, complement 3; C4, complement 4; anti-dsDNA, antibody to double-stranded deoxyribonucleic acid; ESR, erythrocyte sedimentation rate; CI, confidence interval; HR, hazard ratio.

## Data Availability

The study’s original contributions are detailed within the article. Requests for clarification or additional information can be directed to the corresponding author.

## Abbreviations

The following abbreviations are used in this manuscript:

Anti-dsDNA	Antibody to double-stranded deoxyribonucleic acid
C3	Complement 3
C4	Complement 4
CI	Confidence interval
CGRD	Chang Gung Research Database
DMARDs	Disease-modifying antirheumatic drugs
ESR	Erythrocyte sedimentation rate
HR	Hazard ratio
ICD	International Classification of Diseases
IgM	Immunoglobulin M
SARDs	Systemic autoimmune rheumatic diseases
SLE	Systemic lupus erythematosus
